# Exploring Societal Attitudes and Acceptance of Mental Illness in Albania: A Study on Stigma and Treatment Preferences

**DOI:** 10.1192/j.eurpsy.2025.1465

**Published:** 2025-08-26

**Authors:** L. Demiraj, F. Elezi

**Affiliations:** 1 “Mother Teresa” UHC, Tirana, Albania

## Abstract

**Introduction:**

Mental illness remains a significant issue globally, often accompanied by challenges related to stigma, stereotypes, and prejudice. In Albania, research exploring societal perceptions and the acceptance of mental illness and psychiatric treatment is limited. Understanding these views is crucial for improving mental health care and reducing stigma.

**Objectives:**

The aim of this study was to evaluate the perception and prejudice toward mental illness in the Albanian population, with a focus on stigmatization, acceptance, and attitudes toward psychiatric treatment.

**Methods:**

Data collection was carried out through a self-administered online questionnaire distributed to the general population in Albania. A total of 326 questionnaires were analyzed. The study population included individuals born and residing in Albania. The data was processed using the statistical program IMB SPSS v. 23.

**Results:**

The sample showed a significant gender disparity, with a female-to-male ratio of 2.7:1. The majority of respondents were aged 21 to 40 years and had higher education. Religious individuals also showed high participation. While respondents acknowledged high levels of stigma in society, they expressed personal openness toward maintaining relationships with individuals with mental illness and integrating them into society. Despite this, there was a general lack of knowledge regarding mental illness, and confidence in the quality of mental health services in Albania was low. Psychiatrists were recommended for severe cases, though psychologists were preferred for treating depression. The most favored treatment approach was a combination of medication and psychotherapy.

**Conclusions:**

The findings suggest that while there is an awareness of societal stigma toward mental illness in Albania, individuals personally express supportive attitudes toward integration and treatment. However, a gap in knowledge about mental illness and skepticism toward mental health services highlights the need for public education and improvements in the healthcare system. The preference for combined treatment reflects an evolving view on comprehensive care.

**Disclosure of Interest:**

None Declared

